# Prediction of live birth using machine learning based on β-hCG and embryo quality in patients with unexplained recurrent spontaneous abortion undergoing PGT-A

**DOI:** 10.3389/fendo.2026.1898690

**Published:** 2026-08-12

**Authors:** Jiao Lin, Xingping Zhao, Yuxi Chen, Bing Xie, Haiying Cheng, Chen Luo, Xiaoyan Cheng, Juan Liu, Enuo Peng, Xianghong Huang

**Affiliations:** 1 Reproductive Center, Xiangtan Central Hospital, Affiliated Hospital of Hunan University, Xiangtan, Hunan, China; 2 Department of Obstetrics and Gynecology, The Third Xiangya Hospital, Central South University, Changsha, Hunan, China

**Keywords:** immunosuppressant, live birth, machine learning, PGT-A, prediction model, URSA

## Abstract

**Purpose:**

This study aimed to identify key predictors of live birth and to develop a machine learning prediction model for women with unexplained recurrent spontaneous abortion (URSA) undergoing preimplantation genetic testing for aneuploidy (PGT-A).

**Methods:**

This retrospective study analyzed 127 patients with URSA who underwent PGT-A. Patients underwent frozen-thawed embryo transfer (FET) following either a hormone replacement cycle (HRT) or a down-regulated HRT. Data were collected on clinical baseline characteristics, endometrial thickness, embryo quality, medication use, and pregnancy outcomes. Binary logistic regression was used to assess the correlation between these factors and live birth.

**Results:**

Among the 127 patients with URSA, 79 achieved live births and 48 did not. Through comparative and regression analyses, the study identified key predictive variables, providing a reference for evaluating pregnancy outcomes. There were no significant baseline differences between the live birth and non-live birth groups in terms of age, Anti-Müllerian Hormone (AMH), Body Mass Index (BMI), duration of infertility, thyroid-stimulating hormone (TSH), erythrocyte sedimentation rate (ESR), endometrial thickness (EMT), embryo quality, or post-transfer medication (all P>0.05). Univariate regression analysis showed that β-human chorionic gonadotropin (β-hCG) levels on day 14 post-transfer was significantly positively correlated with live birth (P<0.001). Multivariate analysis further identified the day 14 β-hCG level post-transfer and transferred embryo quality as key variables. The constructed random forest machine learning model demonstrated strong predictive performance [Area Under the Curve (AUC)=0.917, 95%CI 0.778-1.000]. The day 14 β-hCG level cut-off for predicting live birth was 457.75 mIU/mL, with an optimal range of 828.69-3571.77 mIU/mL. SHAP analysis showed that high-quality embryos increased the chances of live birth, while low-quality embryos decreased it. Different medications or luteal support regimens did not significantly affect live birth rates.

**Conclusions:**

For URSA patients undergoing PGT-A with euploid embryo transfer, day 14 β-hCG levels and embryo quality were key predictors of live birth. Empirical interventions and luteal support type had no significant impact. The random forest model can accurately predict live births, aiding personalized clinical management and minimizing unnecessary treatments.

## 1 Introduction

Recurrent spontaneous abortion (RSA) is a prevalent pregnancy complication among women of reproductive age. The definition of RSA remains inconsistent across guidelines, with variations predominantly concerning the gestational age at miscarriage, the number of pregnancy losses, whether these losses are consecutive, and the inclusion of biochemical pregnancies. Clinically, RSA is generally characterized by the occurrence of three or more pregnancy losses during early gestation; however, two consecutive pregnancy losses also warrant clinical attention and evaluation, as the risk of subsequent pregnancy loss is similar to that associated with three losses (1). According to the expert consensus in China, RSA is defined as two or more consecutive pregnancy losses before 28 weeks of gestation with the same partner, including biochemical pregnancies (2). The etiological factors contributing to RSA, including chromosomal abnormalities, anatomical defects of the reproductive tract, endocrine disorders, infectious agents, and autoimmune diseases, have been well documented (3). However, a significant proportion of RSA cases remain idiopathic even after thorough clinical evaluation, a condition termed unexplained recurrent spontaneous abortion (URSA). The global incidence of URSA is estimated to range from 1% to 5%, although its true prevalence may be underestimated due to variations in diagnostic criteria, including the number of pregnancy losses and gestational age thresholds (4, 5). The pathogenesis of URSA is complex and multifactorial, potentially encompassing immune regulatory dysfunction, endocrine disturbances, genetic polymorphisms, epigenetic modifications, and environmental and psychological stressors, among other factors (6–10). Nevertheless, the precise etiological mechanisms and underlying pathophysiology remain poorly understood, positioning URSA as a major research focus and a formidable clinical challenge within in assisted reproductive technology.

Embryonic aneuploidy is a significant factor contributing to the failure of assisted reproductive technologies (11). Preimplantation Genetic Testing for Aneuploidy (PGT-A) is employed in patients with URSA to select chromosomally normal euploid embryos for transfer, thereby potentially enhancing clinical pregnancy rates and ongoing pregnancy success (12, 13). Nonetheless, even after the transfer of euploid embryos, the pregnancy loss rate remains elevated at 17.9% (14). Besides embryonic chromosomal status, factors such as the maternal-fetal interface, embryonic quality, imbalanced immune interactions, and the prothrombotic state of the microenvironment significantly influence outcomes. Lin et al. (15) found that high TNF-α and IFN-γ levels, along with low IL-10 levels, significantly raise miscarriage risk in URSA patients (AUC = 0.887). Xu et al. (16) identified coagulation metrics (R, Angle-α, MA) as independent URSA risk factors (AUC = 0.887). Wang et al. (17) created a URSA risk prediction model using standard preconception screening data. The primary limitations of current research concern insufficient data on embryonic ploidy status. Most studies investigating factors influencing live birth outcomes in URSA patients have focused on isolated or limited variables, lacking comprehensive multifactorial analyses. This study retrospectively examined the determinants of live birth outcomes following euploid embryo transfer in URSA patients. The findings offer significant theoretical and clinical value for optimizing decision-making, developing individualized treatment strategies, and enhancing live birth rates within this patient population.

## 2 Materials and methods

### 2.1 Data collection

This retrospective cohort study was conducted at the Reproductive Center of Xiangtan Central Hospital from January 2019 and July 2025, enrolling 127 infertile patients with URSA who underwent PGT-A. Approval was obtained from the Reproductive Ethics Committee of Xiangtan Central Hospital, Hunan University (Approval Number: SZ20260502). All patients underwent frozen embryo transfer (FET) following comprehensive history-guided evaluations and interventions, including hysteroscopy, chromosomal analysis for both partners, screening for antiphospholipid antibody syndrome, antinuclear antibody profiling, panel testing for alloantibodies, rheumatoid factor assessment, thrombophilia screening (comprising antithrombin III, lupus anticoagulant, and anti-glycoprotein antibodies), thyroid function and antibody testing, oral glucose tolerance test with insulin release assay, and serum 25-hydroxyvitamin D measurement. Comprehensive clinical data were collected, including demographic characteristics such as age, weight, body mass index (BMI), duration of infertility, AMH levels, history of adverse pregnancy, live births, endometrial thickness and blood flow, embryo quality and culture days, and post-transplant medication. Inclusion criteria: (1) Age ≤40; (2) Recurrent miscarriage of unknown cause (≥2 first trimester pregnancy loss with at least one normal karyotype embryo); (3) Undergoing PGT-A with at least one euploid embryo; (4) Using hormone therapy for endometrial preparation and undergoing embryo transfer; (5) Euploid single blastocyst transfer; (6) Complete clinical and follow-up data for analysis. Exclusion criteria: (1) The presence of confirmed autoimmune diseases, such as systemic lupus erythematosus or rheumatoid arthritis, or connective tissue diseases; (2) Thin endometrium, characterized by an endometrial thickness of less than 7 mm as measured by ultrasound on the day of embryo transfer; (3) Chromosomal analysis of aborted tissue revealing embryonic chromosomal aneuploidy abnormalities, or chromosomal number or structural abnormalities in either partner; (4) Confirmed coagulation abnormalities or prothrombotic state; (5) A history of recurrent implantation failure, defined as the inability to achieve clinical pregnancy following two or more transfers of high-quality or euploid embryos; (6) Uterine anomalies, including congenital uterine developmental abnormalities, intrauterine adhesions, or uterine fibroids that alter uterine cavity morphology, or other severe developmental abnormalities of the reproductive system; (7) Uncontrolled endocrine disorders (e.g., hypothyroidism/hyperthyroidism, diabetes mellitus, hyperprolactinemia, etc.); (8) The inability to perform embryo transfer due to patient preference or physical condition; (9) Significant gaps in clinical data.

### 2.2 Controlled ovarian hyperstimulation and embryo transfer

All enrolled patients received individualized controlled ovarian hyperstimulation (COH) protocols, with the specific ovulation induction regimen (including GnRH agonist/antagonist protocols, mild stimulation, or luteal phase protocols) tailored according to ovarian reserve markers, baseline hormone profiles, and BMI. Following oocyte retrieval and fertilization via Intracytoplasmic Sperm Injection (ICSI), resulting embryos were cultured to the blastocyst stage before trophectoderm biopsy for PGT-A, and the stages of embryonic biopsy were carefully documented. The Gardner blastocyst grading system was used to evaluate blastocoel expansion, as well as the morphological quality of the inner cell mass (ICM) and trophectoderm (TE) (18). The ICM and TE were each graded as A, B, or C. High-quality blastocysts were defined as those with ICM and TE grades of A or B (AA, AB, BA). Blastocysts with both ICM and TE graded B were defined as medium-quality (BB). Blastocysts with either ICM or TE graded C were categorized as low-quality blastocysts (AC, CA, BC, CB, CC). The biopsy specimens were subjected to next-generation sequencing (NGS) to assess embryo ploidy status, and only euploid embryos were selected for subsequent transfer. Endometrial preparation was performed using either hormone replacement therapy (HRT) or downregulated HRT protocols, with a maximum recommended estrogen dosage of 6 mg/day. The endometrial thickness and blood flow were monitored via transvaginal ultrasound on the day of embryo transfer. For embryo transfer, a single euploid blastocyst was typically transferred under ultrasound guidance, and all transplantation procedures were performed by the same experienced chief physician. All patients received standardized luteal support following frozen-thawed embryo transfer. Beginning on the day of transfer, vaginal progesterone preparations, including Crinone or Utrogestanwere, were administered in combination with oral progesterone. Luteal support was continued in patients with a positive serum β-hCG test at 14 days after transfer. Following confirmation of clinical pregnancy by ultrasound at 6–7 weeks of gestation, the dose was gradually tapered according to routine clinical practice. Luteal support was completely discontinued by 10–12 weeks of gestation in patients with a stable clinical pregnancy. Some patients received adjunctive therapies including cyclosporine, enoxaparin, and prednisone at the discretion of treating physicians, based on clinical experience and after obtaining written informed consent. These medications were administered empirically according to routine clinical practice rather than a standardized study protocol, and the choice and duration of adjunctive treatments were not mandatory or randomized, but dependent on individual clinical judgment and patient preference. All patients were followed up until confirmation of live birth or pregnancy loss.

### 2.3 Reproductive outcomes

The study’s primary endpoints were live birth. Biochemical pregnancy was confirmed by a serum β-hCG levels ≥50 IU/L at 14 days post-transfer, with serial monitoring of β-hCG doubling patterns, while clinical pregnancy was defined as ultrasonographic visualization of an intrauterine gestational sac with fetal cardiac activity at 4–5 weeks post-transfer. Live birth was defined as the delivery of a viable infant after 28 weeks of gestation. First trimester pregnancy loss was defined the pregnancy loss before 12 weeks of gestation. These outcome measures were dichotomized as follows: “YES” representative live birth, “NO” indicates no live birth (miscarriage or embryonic arrest before 28 weeks of pregnancy).

### 2.4 Statistical methods

Statistical analyses were performed using R (version 4.4.1) and Python (version 3.12.0). Continuous variables were presented as mean ± standard deviation, while categorical variables were expressed as frequencies and percentages. Initial descriptive analyses characterized baseline study variables. For comparative analyses, categorical variables were assessed using chi-square tests, and continuous variables were analyzed with Mann-Whitney U tests due to their non-normal distribution. Univariate logistic regression was initially performed for all candidate variables. Variables yielding P<0.5 in the univariate analysis were entered into backward stepwise multivariable logistic regression. The logistic regression model incorporated three predictors (df = 3). A total of 48 non-live birth events were recorded in this cohort, and the events per variable (EPV) was calculated as 48/3 = 16. This value exceeds the widely recognized EPV threshold of 10 recommended in statistical guidelines, demonstrating that the number of model parameters satisfies the statistical requirements for predictive modeling with small sample sizes. The dataset was divided into a training set (70%) and an independent test set (30%) at a ratio of 7:3. Stratified sampling (stratify=y) was applied to maintain balanced outcome distribution between the two subsets, and random_state=42 was fixed to ensure experimental reproducibility. Nine machine learning models were established, and all models included the same three predictors. Notably, bootstrap internal validation with 1,000 resamples was performed only on the optimal random forest model to evaluate model calibration.

## 3 Result

### 3.1 Baseline clinical characteristics of study subjects

A total of 127 women with URSA undergoing euploid embryo transfer following PGT-A were included in this study after strict inclusion and exclusion criteria were applied. Comparative analyses demonstrated a significant association between live birth and β-hCG levels at day 14 post-transfer, with the live birth group showing markedly higher β-hCG levels than the non-live birth group (*P* < 0.001). Baseline characteristics including age, weight, BMI, BMI classification, number of adverse pregnancy, live birth and induced abortion, duration of infertility, basal hormone levels, thyroid-stimulating hormone (TSH), ESR, EMT at ultrasound day, subendometrial blood flow patterns, embryo quality and culture days, empirical medications (Crinone/Utrogestan, Ciclosporin, Prednison, Enoxaparin), had no statistically significant impact on outcomes (All *P*>0.05), indicating a highly homogeneous study population with minimal confounding bias, as detailed in **Table 1**.

**Table 1:** Baseline characterization of study variables.

Clinical characteristics	Live birth		Statistic	*P*
	No (n=48)	Yes (n=79)		
Number of RSA			χ²=0.099	0.952
2	34 (70.8%)	58 (71.0%)		
≥3	14(29.2%)	21 (29.0%)		
Number of induced abortion			χ²=3.19	0.363
0	38 (79.2%)	68 (86.1%)		
1	6 (12.5%)	7 (8.9%)		
2	3 (6.2%)	1 (1.3%)		
3	1 (2.1%)	3 (3.8%)		
Number of live births			χ²=2.37	0.500
0	17 (35.4%)	23 (29.1%)		
1	23 (47.9%)	44 (55.7%)		
2	7 (14.6%)	12 (15.2%)		
3	1 (2.1%)	0 (0.0%)		
embryo quality			χ²=3.84	0.147
BA+AB	2 (4.2%)	1 (1.3%)		
BB	25 (52.1%)	54 (68.4%)		
BC	21 (43.8%)	24 (30.4%)		
Embryo culture days			χ²=2.44	0.296
Day5	28 (58.3%)	49 (62.0%)		
Day6	17 (35.4%)	29 (36.7%)		
Day7	3 (6.2%)	1 (1.3%)		
Luteal support			χ²=0.16	0.686
Utrogestan	12 (25.0%)	16 (20.3%)		
Crinone	36 (75.0%)	63 (79.7%)		
Cyclosporine			χ²=0.02	0.902
(-)	22 (45.8%)	34 (43.0%)		
(+)	26 (54.2%)	45 (57.0%)		
Enoxaparin			χ²=2.29	0.130
(-)	25 (52.1%)	29 (36.7%)		
(+)	23 (47.9%)	50 (63.3%)		
Prednisone			χ²=0.02	0.884
(-)	33 (68.8%)	52 (65.8%)		
(+)	15 (31.2%)	27 (34.2%)		
BMI classification (Kg/m^2^)			χ²=1.03	0.794
<18.5	3 (6.2%)	8 (10.1%)		
18.5-24	32 (66.7%)	52 (65.8%)		
24-28	9 (18.8%)	15 (19.0%)		
≥28	4 (8.3%)	4 (5.1%)		
BMI (Kg/m^2^)	22.34 ± 3.99	22.27 ± 3.17	t=-0.098	0.922
Weight (Kg)	56.29 ± 8.92	56.54 ± 8.86	t=0.155	0.877
Duration of infertility (year)	2.03 ± 1.33	2.36 ± 1.65	t=1.202	0.232
Basal FSH (mIU/mL)	6.27 ± 1.82	6.26 ± 1.78	t=-0.045	0.964
Basal LH (mIU/mL)	5.08 ± 2.70	5.06 ± 2.75	t=-0.031	0.975
TSH (mIU/L)	1.98 ± 1.05	2.31 ± 1.27	t=1.515	0.132
ESR (mm/h)	9.27 ± 7.31	9.85 ± 6.29	t=0.470	0.639
EMT on ultrasound day (mm)	9.19 ± 1.66	9.72 ± 1.97	t=1.571	0.119
Arteriae uterina PI	2.45 ± 0.66	2.45 ± 0.77	t=0.037	0.971
Age squared	1148.75 ± 257.40	1148.54 ± 235.83	t=-0.005	0.996
Age*AMH	145.42 ± 143.83	148.10 ± 117.36	t=0.114	0.909
AMH (ng/ml)	2.95 (2.17, 4.72)	3.59 (2.26, 5.79)	U=2107.0	0.295
Arteriae uterina S/D ratio	6.43 (5.15, 7.44)	6.70 (5.13, 8.25)	U=2040.5	0.474

RSA, Recurrent spontaneous abortion, BMI, Body Mass Index, AMH, Anti-Müllerian Hormone, FSH, Follicle-Stimulating Hormone, LH, Luteinizing Hormone, TSH: Thyroid-stimulating hormone, ESR, Erythrocyte sedimentation rate, EMT, Endometrial thickness, PI, pulsatility index, S/D, Systolic/Diastolic ratio.

### 3.2 Univariate logistic regression analysis of correlates

Logistic regression identified β-hCG levels on day 14 post-transfer as a significant predictor of live birth in women with URSA, emerging as an independent protective factor (OR = 1.002, 95% CI 1.001-1.003, *P*<0.001). All other variables, including age, BMI, duration of infertility, ovarian reserve markers [AMH, basal follicle-Stimulating Hormone (FSH)/Luteinizing Hormone (LH)], thyroid function, endometrial thickness, uterine artery blood flow [pulsatility index (PI), Systolic/Diastolic (S/D) ratio], number of induced abortion and live births, embryo quality, and empirical medications, showed no statistically significant association with live birth outcomes (all *P* > 0.05). Variables with *P* < 0.5, namely infertility duration, BMI classification, number of induced abortion and live births, TSH level, endometrial thickness, embryo quality, embryo culture days, enoxaparin administration, and β-hCG levels at day 14 post-transfer, were selected using the backward Wald method. These variables were then entered together into a multivariate Logistic regression model for further statistical analysis, as detailed in **Table 2**.

**Table 2:** Univariable logistic regression analysis of factors associated with live birth.

Variables	β	S.E	Z	OR (95%CI)	*P*
Age	0.003	0.05	0.063	1.00 (0.91-1.11)	0.95
BMI	-0.005	0.053	-0.099	0.99 (0.90-1.11)	0.921
Weight	0.003	0.021	0.156	1.00 (0.96-1.05)	0.876
Duration of infertility	0.152	0.128	1.193	1.16 (0.92-1.52)	0.233
AMH	0.008	0.048	0.171	1.01 (0.92-1.12)	0.864
Basal FSH	-0.005	0.103	-0.045	1.00 (0.81-1.22)	0.964
Basal LH	-0.002	0.068	-0.031	1.00 (0.87-1.15)	0.975
TSH	0.248	0.166	1.494	1.28 (0.94-1.81)	0.135
ESR	0.013	0.028	0.473	1.01 (0.96-1.07)	0.636
EMT on ultrasound day	0.161	0.104	1.553	1.17 (0.96-1.45)	0.12
Arteriae uterina PI	0.009	0.251	0.037	1.01 (0.62-1.67)	0.97
Arteriae uterina S/D	-0.014	0.046	-0.296	0.99 (0.90-1.09)	0.767
Age squared	0	0.001	-0.005	1.00 (1.00-1.00)	0.996
Age*AMH	0.03	0.073	0.42	1.03 (0.89-1.19)	0.675
β-hCG levels at day 14 post-transfer (mIU/mL)	0.002	0	5.044	1.00 (1.00-1.00)	<0.001
Number of RSA					
2				1.00 (Reference)	
≥3	-0.1286	0.407	-0.316	0.8793 (0.40-1.95)	0.752
Number of induced abortion					
0				1.00 (Reference)	
1	-0.428	0.592	-0.723	0.65 (0.20-2.16)	0.47
2	-1.681	1.172	-1.434	0.19 (0.01-1.51)	0.152
3	0.517	1.172	0.441	1.68 (0.21-34.53)	0.659
Number of live births					
0				1.00 (Reference)	
1	0.344	0.406	0.847	1.41 (0.63-3.13)	0.398
2	0.216	0.559	0.386	1.24 (0.42-3.84)	0.698
3	-1.393	1.663	-0.838	0.25 (0.00-4.94)	0.365
Embryo quality					
BA+AB				1.00 (Reference)	
BB	1.463	1.248	1.172	4.32 (0.40-95.55)	0.241
BC	0.827	1.261	0.656	2.29 (0.20-51.28)	0.512
Embryo culture days					
5				1.00 (Reference)	
6	-0.026	0.387	-0.066	0.97 (0.46-2.10)	0.947
7	-1.658	1.179	-1.407	0.19 (0.01-1.57)	0.159
Luteal_support					
Utrogestan				1.00 (Reference)	
Crinone	0.272	0.435	0.625	1.31 (0.55-3.07)	0.532
Cyclosporine					
(-)				1.00 (Reference)	
(+)	0.113	0.368	0.308	1.12 (0.54-2.31)	0.758
Enoxaparin					
(-)				1.00 (Reference)	
(+)	0.628	0.371	1.691	1.87 (0.91-3.91)	0.091
Prednisone					
(-)				1.00 (Reference)	
(+)	0.133	0.391	0.34	1.14 (0.53-2.50)	0.734
BMI classification(Kg/m^2^)					
<18.5				1.00 (Reference)	
18.5-24	-0.495	0.713	-0.694	0.61 (0.13-2.28)	0.487
24-28	-0.47	0.798	-0.589	0.63 (0.11-2.83)	0.556
≥28	-0.981	0.979	-1.002	0.38 (0.05-2.50)	0.316

BMI, Body Mass Index, AMH, Anti-Müllerian Hormone, FSH, Follicle-Stimulating Hormone, LH, Luteinizing Hormone, TSH, Thyroid-stimulating hormone, ESR, Erythrocyte sedimentation rate, EMT, Endometrial thickness, PI, pulsatility index, S/D, Systolic/Diastolic ratio, RSA, Recurrent spontaneous abortion.

### 3.3 Independent predictors of live birth: multivariate logistic regression analysis

The screened variables were incorporated into a multivariate adjusted logistic regression model to control for potential confounders. The final analysis identified serum β-hCG levels and embryo quality as significant predictors of live birth outcomes. Notably, serum β-hCG level emerged as the primary predictor with a statistically significant influence on the outcome (importance ≈ 0.8), whereas embryo quality acted as a secondary predictor with a relatively smaller influence (importance ≈ 0.2). β-hCG levels at day 14 post-transfer were found to be an independent protective factor for live birth (β=0.002, OR = 1.002, 95% CI: 1.001-1.003, *P*<0.001); specifically, each 1.0 mIU/mL increase in early post-transfer serum β-hCG concentration was associated with a 0.2% higher likelihood of live birth. The quality of transferred embryos was also independently linked to live birth outcomes (*P* < 0.05), despite abnormally high odds ratios (up to 3369) in certain subgroups, which were likely attributable to the small sample size of the reference group. In line with clinical relevance and the subsequent SHAP analysis, higher embryo quality corresponded to more favorable outcomes. After adjustment, none of the other variables in the model showed an independent statistical association with live birth (All *P* > 0.05) as detailed in **Table 3**.

**Table 3:** Multivariable logistic regression analysis of factors associated with live birth.

Variables	β	SE	Wald	df	Sig.	OR (95%)
Day 14 Post-Transfer β-hCG (mIU/mL)	0.002	0	25.819	1	0	1.002 (1.001-1.003)
Embryo quality			7.589	2	0.022	
BB or above	4.652	1.771	6.903	1	0.009	104.823 (3.26-3369.991)
BC or lower	4.139	1.788	5.356	1	0.021	62.72 (1.884-2087.483)

### 3.4 SHAP analysis of key predictive factors

**Figure 1:**
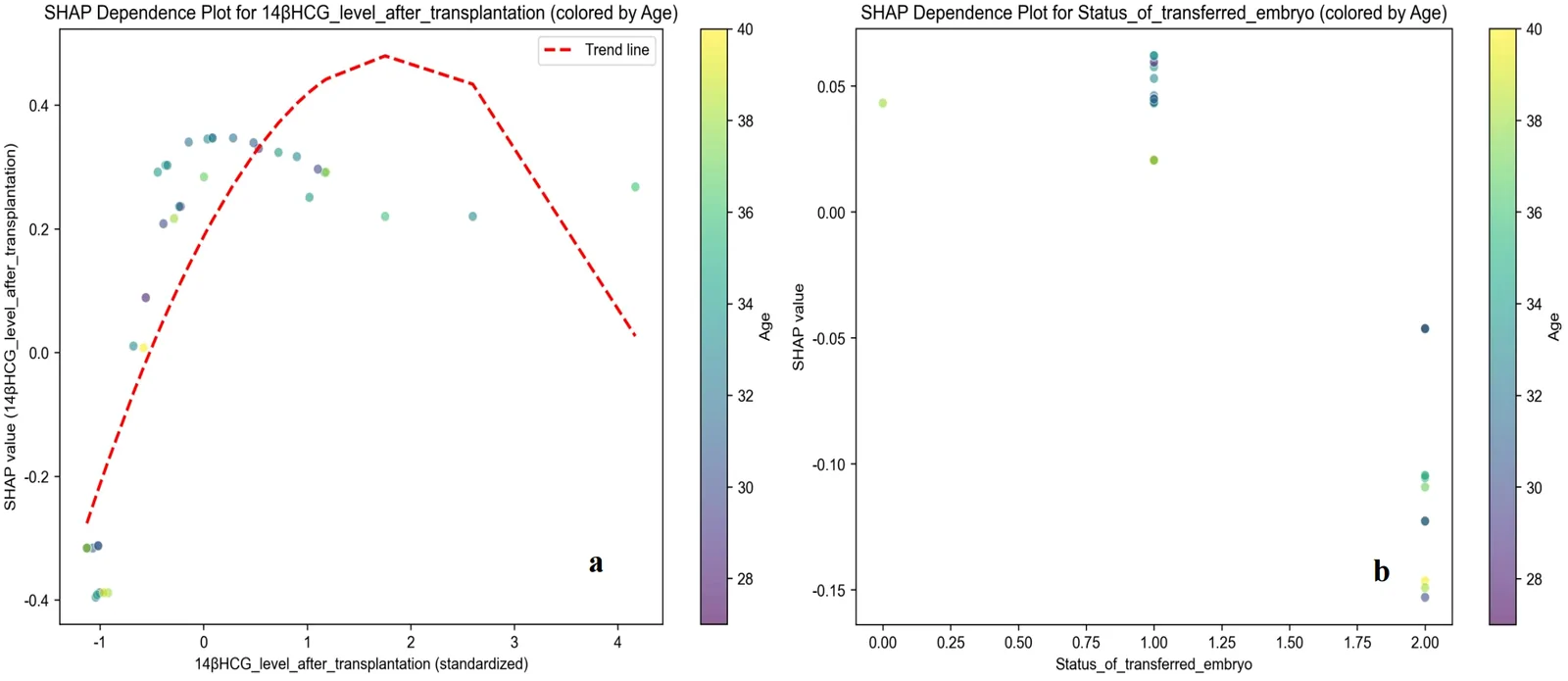
SHAP analysis of the impact of 14β-hCG level after transplantation **(a)** and embryo quality **(b)** on pregnancy outcomes.

This study clarified the nonlinear link between β-hCG levels at day 14 post-transfer and live birth outcomes using SHAP dependency graphs. A threshold effect was identified: β-hCG levels at this time point predict live birth, with a key threshold at 457.75 mIU/mL. Levels between 828.69 and 3571.77 mIU/mL were associated with higher live birth chances, but this benefit plateaued beyond 3571.77 mIU/mL. These results indicate that β-hCG levels reflect early trophoblastic function and embryonic development, with higher levels within a certain range suggesting better embryo implantation and reduced pregnancy loss risk. Beyond a certain threshold, the predictive value for live birth outcomes no longer showed significant enhancement. This may be due to a reduced sample size, potential risks from excessive trophoblastic cell proliferation, or other confounding variables. This finding highlights that β-hCG levels measured on day 14 after embryo transfer serve as a fundamental predictor of pregnancy outcomes (**Figure 1a**).

The SHAP dependence plot quantifies the marginal predictive contribution of embryo transfer status to live birth probability. In the present analysis, higher standardized values of this feature correspond to lower embryo quality. High-quality embryos (grade BB or above) make a more substantial positive contribution to the likelihood of live birth. By contrast, progressive deterioration in embryo quality (grade BC or lower) produces a gradually worsening adverse impact on live birth odds. Poor-quality embryos correspond to negative SHAP values, which denotes a considerable decrease in the model-predicted probability of live birth (**Figure 1b**).

### 3.5 Construction and performance of machine learning prediction model

**Figure 2:**
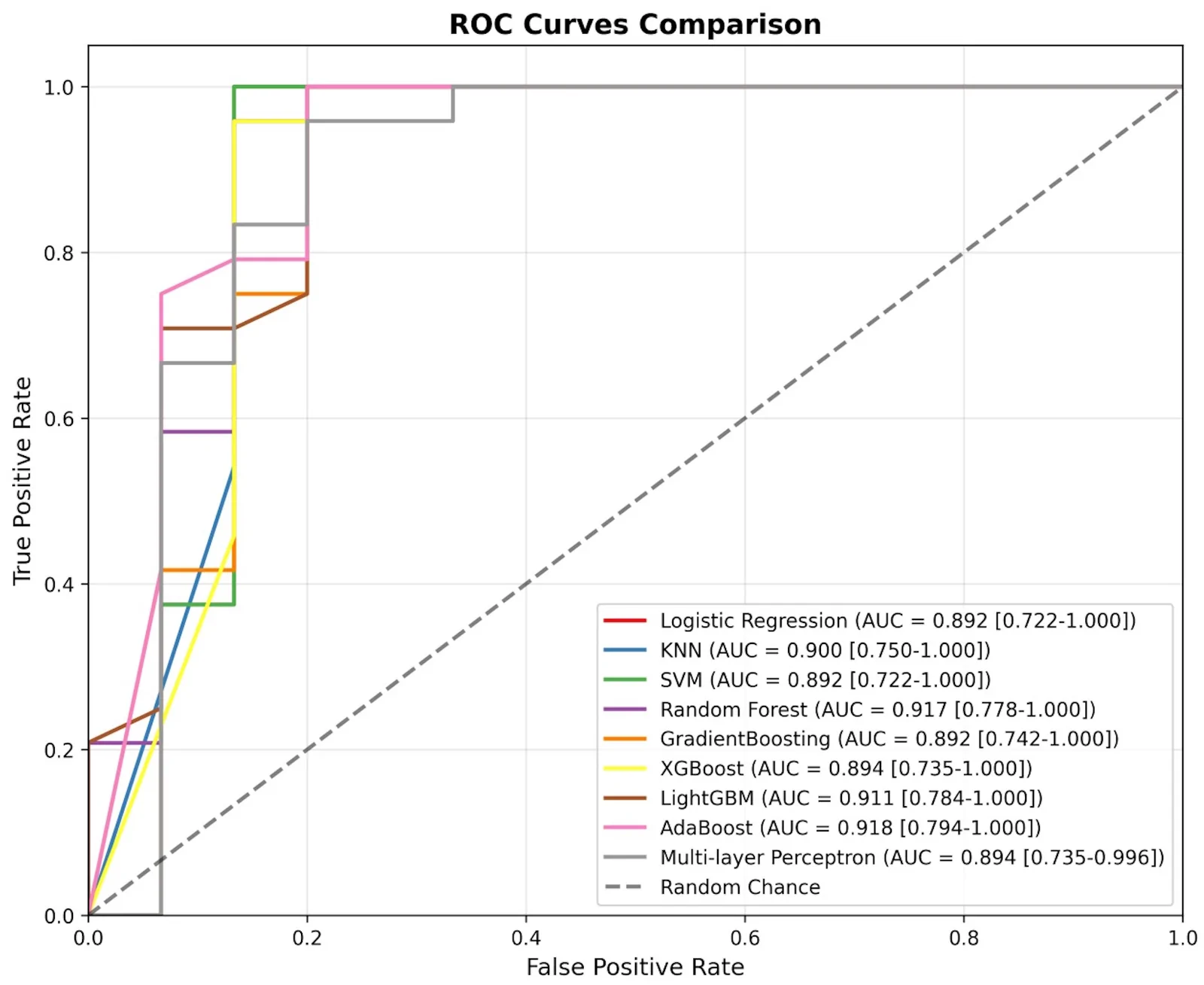
The AUC of different prediction model for URSA.

Nine machine learning algorithms were trained using the selected predictive factors: Logistic Regression (AUC = 0.892, 95%CI 0.722-1.000), Support Vector Machine (SVM) (AUC = 0.892, 95%CI 0.722-1.000), K-Nearest Neighbors (KNN) (AUC = 0.900, 95%CI 0.750-1.000), Gradient Boosting (AUC = 0.892, 95%CI 0.742-1.000), Random Forest (AUC = 0.917, 95%CI 0.778-1.000), LightGBM (AUC = 0.911, 95%CI 0.784-1.000), XGBoost (AUC = 0.894, 95%CI 0.735-1.000), AdaBoost (AUC = 0.918, 95%CI 0.794-1.000), and Multilayer Perceptron (AUC = 0.894, 95%CI 0.735-0.996) (**Figure 2**).

**Figure 3:**
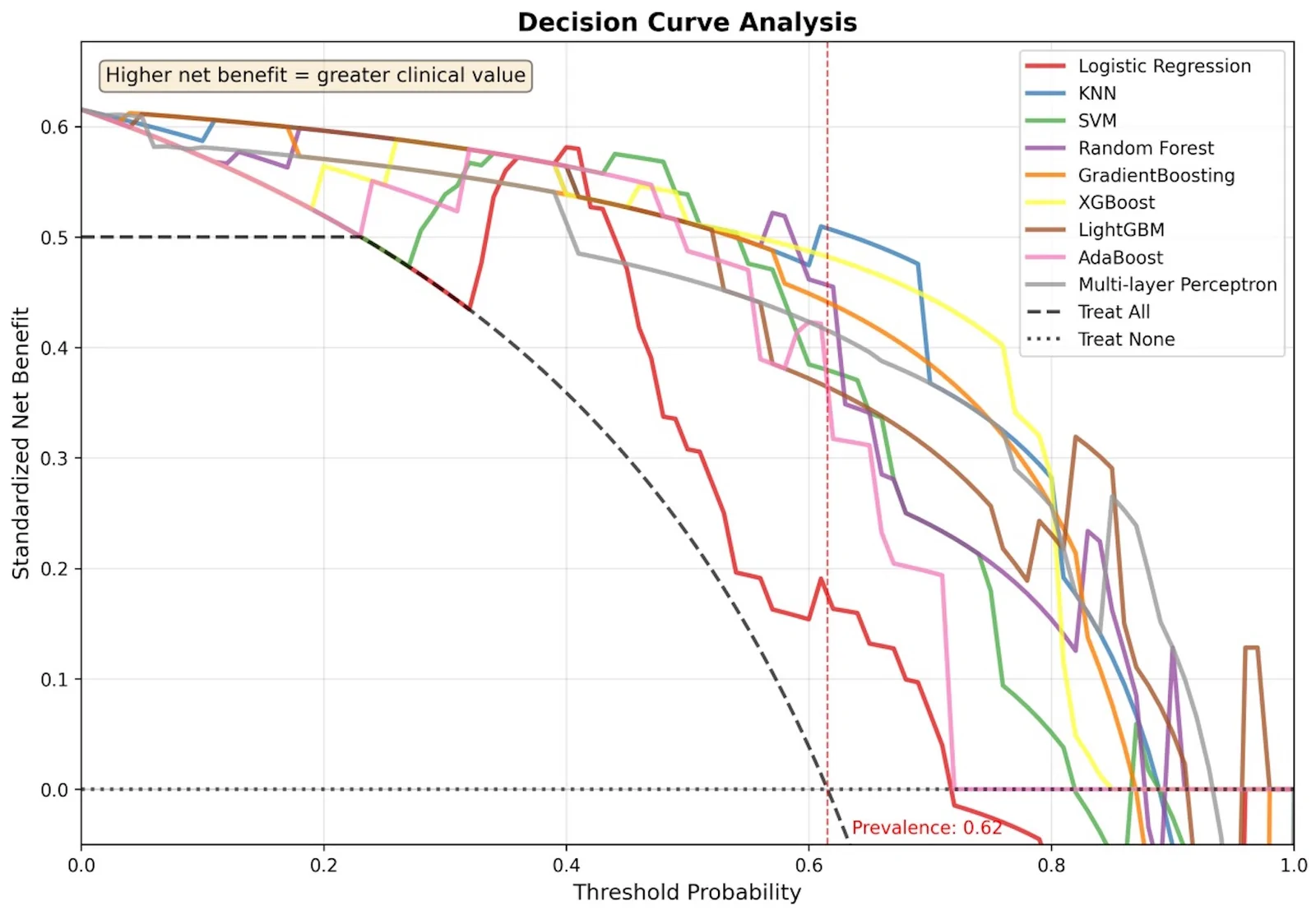
DAC curve of different prediction model for URSA.

Decision curve analysis was used to assess the standardized net benefit of each model across threshold probabilities and compare clinical utility. Models with higher net benefits correspond to better clinical value. LightGBM attained the highest net benefit for most thresholds, particularly 0–0.8, showing optimal clinical performance. XGBoost, GradientBoosting and AdaBoost ranked second, followed by KNN, random forest and multi-layer perceptron, which exceeded logistic regression and SVM. From threshold 0 to 0.6, ensemble models (LightGBM, XGBoost) presented markedly higher net benefits than treat-all and treat-none strategies. In general, LightGBM, XGBoost and GradientBoosting outperformed logistic regression and SVM, especially at low-to-moderate thresholds (**Figure 3**).

**Figure 4:**
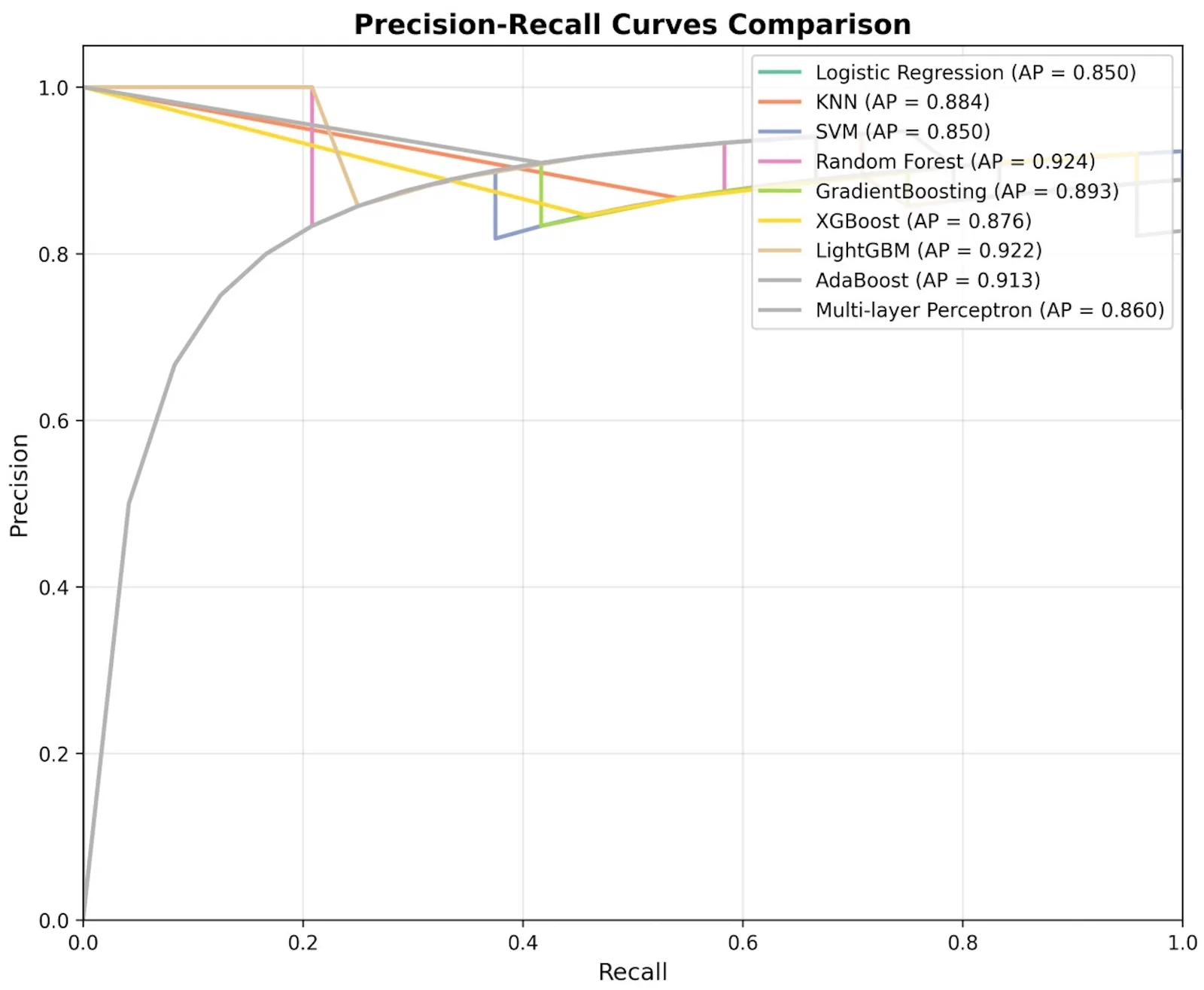
Precision-recall curves for different models.

The precision-recall curve (PRC) was applied to compare the predictive performance of classification models, and Average Precision (AP) was used to quantify overall discriminative ability, where higher AP represents better performance. Random Forest (AP = 0.924) and LightGBM (AP = 0.922) performed optimally, maintaining high precision over all recall values and having the largest area under the PRC. AdaBoost (AP = 0.913) and GradientBoosting (AP = 0.893) achieved suboptimal results. KNN (AP = 0.884) and XGBoost (AP = 0.876) delivered above-average performance, with high precision at low recall and substantial precision reduction when recall increased. The Multi-layer Perceptron (AP = 0.860) gained precision rapidly at recall <0.2, followed by an obvious precision drop. Logistic regression (AP = 0.850) and SVM (AP = 0.850) exhibited relatively poor performance. Their precision was high merely at low recall and declined sharply afterwards, contributing to a small area under the PRC (**Figure 4**).

The random forest model passed the Hosmer–Lemeshow (HL) test, with a P-value > 0.05 (P = 0.14), as detailed in **Table 4** indicating favorable agreement between predicted probabilities and observed event risks, which supports its utility for clinical risk stratification. This model showed excellent discriminative ability with an AUC of 0.917 (95% CI 0.778-1.000), alongside strong overall predictive accuracy (Accuracy = 0.897, F1-score = 0.896), comparable to LightGBM and KNN models. It maintained balanced precision and recall, with a stable confidence interval for AUC (lower bound = 0.778) free from severe boundary attenuation. Therefore, the random forest was selected as the final machine learning prediction model.

**Table 4:** Hosmer-Lemeshow test for different models.

Model	Accuracy	Precision	Re-call	F1-score	ROC- AUC	AUC_CI_ lower	AUC_CI_ upper	HL_P value	Confusion matrix
KNN	0.897	0.899	0.897	0.896	0.9	0.75	1	0.15	[[12 3] [ 1 23]]
LightGBM	0.897	0.899	0.897	0.896	0.911	0.784	1	0.08	[[12 3] [ 1 23]]
Random Forest	0.897	0.899	0.897	0.896	0.917	0.778	1	0.14	[[12 3] [ 1 23]]
Gradient Boosting	0.897	0.899	0.897	0.896	0.892	0.742	1	0.03	[[12 3] [ 1 23]]
XGBoost	0.897	0.897	0.897	0.897	0.894	0.735	1	0.04	[[13 2] [ 2 22]]
AdaBoost	0.872	0.871	0.872	0.871	0.918	0.794	1	0.00	[[12 3] [ 2 22]]
SVM	0.872	0.874	0.872	0.872	0.892	0.722	1	0.01	[[13 2] [ 3 21]]
Multi-layer Perceptron	0.846	0.846	0.846	0.846	0.894	0.735	0.996	0.00	[[12 3] [ 3 21]]
Logistic Regression	0.692	0.756	0.692	0.694	0.892	0.722	1	0.00	[[13 2] [10 14]]

**Figure 5:**
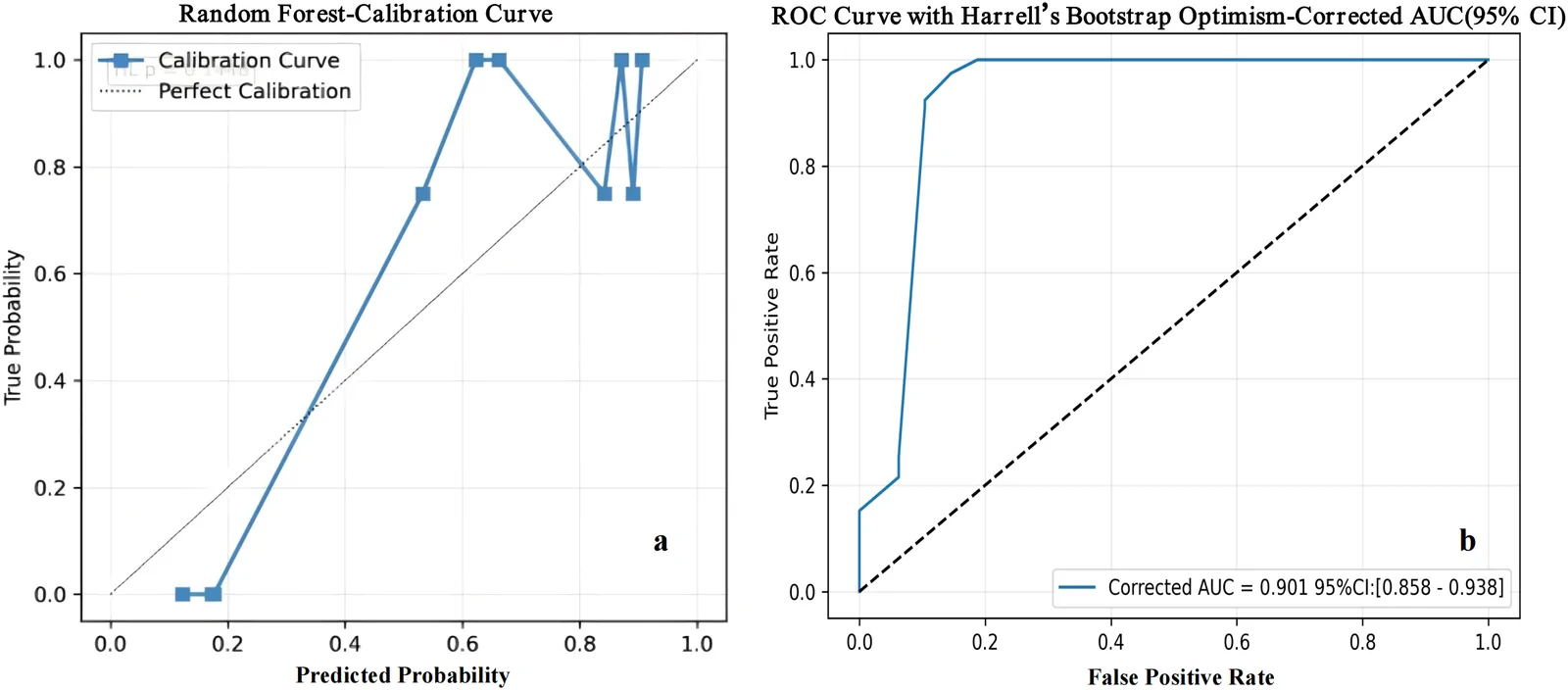
Calibration curve **(a)** and internal validation of prediction model **(b)** of the random forest model.

The calibration curve of the random forest model demonstrated good consistency between predicted probabilities and observed event risks, reflecting favorable calibration. Predictions matched actual event rates well in low- and moderate-risk strata. Minor fluctuations in the high-risk stratum stemmed from uneven sample sizes without evident systematic bias, confirming reliable predictive performance (**Figure 5a**). Bootstrap internal validation (1,000 resamples) was implemented for the random forest model. The apparent C-statistic of the random forest model was 0.917 (95%CI 0.778-1.000), and the optimism-corrected C-statistic was 0.901(95%CI 0.858-0.938). The small difference indicates minimal model pessimism, and the bias-adjusted calibration curve shows acceptable agreement between predicted risks and observed outcomes. These results verify the low overfitting risk of this parsimonious model (**Figure 5b**).

### 3.6 Efficacy of different clinical intervention regimens

**Figure 6:**
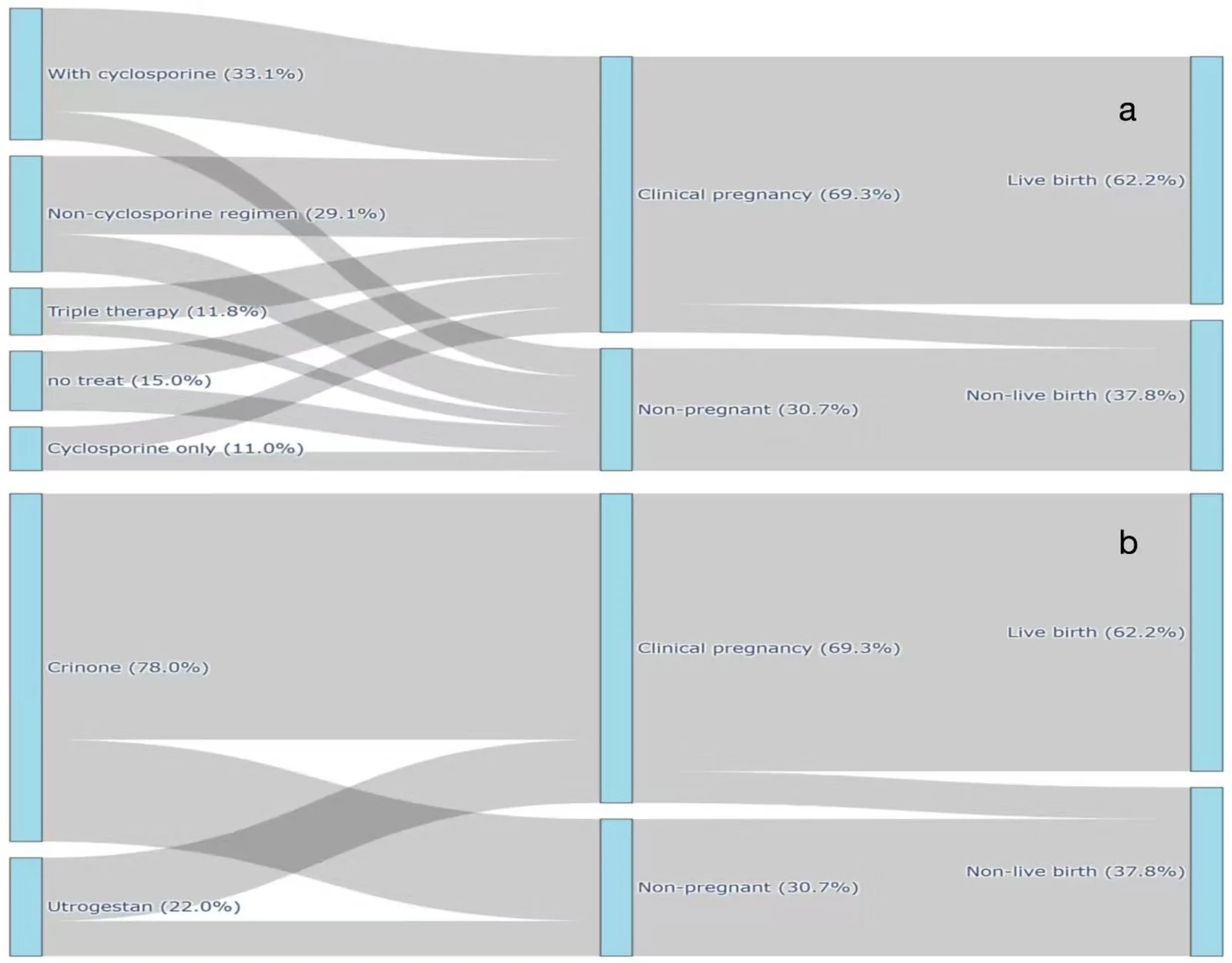
Sankey diagram of immunosuppressants **(a)**, luteal support medications**(b)** and pregnancy outcomes following embryo transfer.

In the subgroup analysis of immunotherapeutic regimens, no significant intergroup differences were detected in the distribution of pregnancy outcomes among the cyclosporine-containing regimen group, non-cyclosporine regimen group, triple immunotherapy group, cyclosporine monotherapy group and non-immunotherapy group. Clinical pregnancy rates and live birth rates were comparable across all groups, and no immunotherapeutic regimens conferred a distinct beneficial advantage in terms of pregnancy outcomes. Collectively, these findings indicate that empirical immunosuppressive therapy fails to improve pregnancy outcomes in patients with URSA lacking confirmed immunological abnormalities. Blind administration of immunotherapy not only fails to elevate live birth rates, but may also elevate the risk of drug-related adverse reactions and augment medical economic burdens. (**Figure 6a**).

Analysis of luteal support strategies showed similar clinical pregnancy and live birth rates between Crinone and Utrogestan groups, indicating equivalent efficacy of the two vaginal progesterone formulations. The higher clinical preference for Crinone stemmed from easier administration and better patient compliance rather than therapeutic superiority. After PGT-A screening and exclusion of major miscarriage risks, both agents effectively maintained endometrial receptivity and supported pregnancy progression. Therefore, individualized luteal support protocols can be determined according to patients’ tolerance, preference, and economic status (**Figure 6b**).

## 4 Discussion

Recent global epidemiological data indicate that approximately 23 million spontaneous abortions occur annually worldwide, while RSA affects between 0.5% and 5% of reproductive couples (19). This condition imposes significant emotional and psychological burdens on affected couples, potentially leading to a range of mental health disorders, including post-traumatic stress disorder (PTSD), anxiety, depression, and even suicidal ideation (20). For women diagnosed with RSA, comprehensive diagnostic evaluations and implement targeted therapeutic interventions are essential, guided by a thorough collection of medical history (21). However, the underlying etiology remains unidentified in approximately 40% of RSA cases, which are categorized as unexplained RSA (22). The advancement and application of artificial intelligence technology enhance the efficiency of screening high-risk RSA populations, facilitate the stratified clinical management of RSA patients, provide robust evidence for individualized treatment decision-making, and ultimately optimize pregnancy outcomes for these patients.

This study evaluated the clinical applicability of machine learning algorithms in predicting pregnancy outcomes associated with assisted reproductive technology by developing various machine learning prediction models. Jian et al. (23) conducted a systematic review and critical appraisal of existing risk prediction models for pregnancy outcomes in cases of RSA. Their analysis identified 65 independent predictive variables, which were organized into five principal categories: (1) patient-related factors, encompassing maternal and paternal age, BMI, and the number of spontaneous abortions; (2) imaging-related factors, such as mean gestational sac diameter and biparietal diameter; (3) thrombophilia-related factors, including D-dimer and protein S levels; (4) metabolic and endocrine-related factors, such as progesterone, TSH, and AMH; and (5) immunological factors, including antinuclear antibody (ANA), anti-cardiolipin antibody (ACA), and complement 4 (C4). This study was exclusively concentrated on URSA. Comprehensive preoperative baseline assessments were conducted to meticulously exclude thrombotic, metabolic, endocrine, and immunological factors associated with recurrent spontaneous abortion. Additionally, we incorporated critical variables directly related to pregnancies conceived through ART, such as embryo quality, β-hCG levels at day 14 post-transfer, and post-transfer medication regimens. Predictors identified by regression analysis (embryo quality and β-hCG levels at day 14 post-transfer) were used to develop machine learning models. The random forest model exhibited favorable predictive performance (AUC = 0.917, 95%CI 0.778-1.000) and passed the HL test, suggesting adequate calibration. The overall accuracy reached 0.897 with an F1 score of 0.896. The balanced precision and recall achieved illustrates the reliable fitting capacity of the random forest model. Bootstrap internal validation with 1,000 resampling iterations further verified that the model carries a low risk of overfitting. However, the random forest model employed in this study had a small sample size, which compromised its stability and generalization performance. To address the issue of small-sample bias, this study adopted repeated hierarchical cross-validation and parameter grid optimization methods. Further research should enroll multi-center large-sample cohorts to expand the sample size. Such efforts can stabilize model training, narrow confidence intervals for subgroup analyses, and validate and refine the stratified predictive capacity of the model.

β-hCG level day 14 post-transfer emerged as the most significant predictor of final pregnancy outcomes. As a placenta-derived glycoprotein, this biomarker is practical and economically efficient, and its dynamic fluctuations serve as a fundamental indicator for evaluating embryonic viability. It promotes embryonic implantation and angiogenesis, regulates trophoblast cell development, and plays a pivotal role in modulating the maternal-fetal interface immune response throughout pregnancy (24). Research indicates that β-hCG levels measured 14 days post-transfer are the most accurate predictor of live birth (25). Dhar et al. (26) identified a cutoff value of 468 mIU/mL for this indicator as the optimal threshold for predicting live birth outcomes, with an AUC of 0.788. Concurrently, Zhang et al. (27) confirmed that its serum levels measured on day 17 after oocyte retrieval could effectively predict clinical pregnancy outcomes in IVF/ICSI cycles, with levels in patients who achieved live birth being significantly higher than those in miscarriage cases (596.8 mIU/mL vs. 357.15 mIU/mL, *P* < 0.001). The present study further quantified the predictive value of this marker using machine learning approaches and uncovered a nonlinear threshold effect on live birth outcomes. Specifically, levels below 457.75 mIU/mL were linked to a markedly reduced likelihood of live birth, which aligns with the findings of Dhar et al. (26). Additionally, our analysis revealed that the probability of live birth was highest when β-hCG levels ranged from 828.69 to 3571.77 mIU/mL, with its predictive efficacy diminishing beyond this range. These findings offer a quantitative reference for clinical risk stratification in early pregnancy, enabling clinicians to promptly identify high-risk populations and implement targeted modifications to clinical intervention strategies.

Embryo selection is crucial in IVF. This study used the Gardner blastocyst grading system to evaluate blastocoel expansion and the morphology of the ICM and TE. Compared with high-quality blastocysts, live birth rates were significantly lower in medium- and low-grade groups (low-grade: adjusted odds ratio [aOR] = 0.48; medium-grade: aOR = 0.70). ICM quality was a key factor, with grade B or C ICM blastocysts showing much lower live birth rates than grade A, similar to TE grading results (28). In euploid embryos, ICM grading independently predicts live birth outcomes for day 5 blastocysts (29). Live birth rates were significantly higher for day 5 blastocysts than for day 6 blastocysts (62.1% vs. 53.6%) (30). Embryo quality is the key factor in successful transfers, with embryos graded BB or higher significantly improving pregnancy outcomes in patients with URSA. As embryo quality diminishes to BC or lower, its adverse effect on pregnancy outcomes intensifies significantly. However, the length of embryo culture (Day 5/6/7) demonstrated no statistically significant association with live birth outcomes (aOR = 0.97, *P* = 0.947). This insight enables clinicians to more effectively communicate the pregnancy risks associated with varying embryo qualities prior to embryo transfer, thereby assisting patients in making more informed decisions regarding their embryo transfer strategies.

The effect of adjuvant therapy on pregnancy outcomes after embryo transfer remains a major clinical issue. Despite numerous attempts at miscarriage prevention, no specific treatment has been proven effective. Immunosuppressive and anticoagulant therapies are commonly used for unexplained recurrent miscarriage. Cyclosporine works inhibits T-cell differentiation, alters cytokine levels, reduces NK cell activity, restores maternal-fetal immune tolerance, and promotes trophoblast growth, embryo implantation, and placental development. In women with recurrent spontaneous abortion, cyclosporine reduced miscarriage rates by 63% and increased live birth and sustained pregnancy rates by 1.44 and 1.59 times, respectively (31). In cases of refractory unexplained recurrent spontaneous abortion, cyclosporine significantly improved the live birth rate from 59.0% to 74.3% (32). The analysis of immune intervention regimens revealed no significant differences in pregnancy outcomes, including clinical pregnancy and live birth rates, among the cyclosporine-containing, non-cyclosporine, triple immunotherapy, cyclosporine monotherapy, and non-immune intervention groups. No immune intervention regimen exhibited a clear advantage in improving pregnancy outcomes. Combined with the strict inclusion criteria of this study, which excluded some confounding factors related to immune abnormalities, these results confirm that empirical use of immunosuppressants fails to improve pregnancy outcomes in patients with URSA without definite immune abnormalities. Blind immune intervention not only fails to elevate the live birth rate, but also increase the risk of drug-related adverse reactions and medical costs. Further large-scale randomized controlled trials are warranted to validate the scientific plausibility and reliability of these hypotheses.

Luteal support plays a critical role in sustaining pregnancy during ART, yet the predictive value of serum progesterone remains controversial. A large-scale study by Coomarasamy et al (33). demonstrated no significant difference in live birth rates between progesterone and placebo administration. Nonetheless, clinical guidelines recommend progesterone supplementation for women with early pregnancy bleeding and a history of pregnancy loss. Different vaginal progesterone preparations have been shown to achieve comparable live birth rates and safety profiles. The analysis of luteal support regimens revealed that the conversion ratios of clinical pregnancy, non-pregnancy and live birth outcomes were highly consistent between Crinone group and Utrogestan group, directly verifying that the two progesterone preparations have equivalent luteal support effects in this population (34, 35). On the premise that embryos were screened as euploid via PGT-A and all known risk factors for miscarriage were excluded, both luteal support drugs can effectively maintain endometrial receptivity and ensure continuous pregnancy progression. Clinically, individualized selection of luteal support regimens can be implemented based on patients’ individual tolerance, medication habits and economic conditions, without being restricted to a single dosage form.

Several limitations of this study should be acknowledged. First, this was a single-center retrospective study. First, This study adopted strict exclusion criteria to obtain a homogeneous cohort, which inevitably narrowed the study population. Accordingly, our conclusions are only applicable to patients with unexplained recurrent spontaneous abortion (URSA) without thin endometrium, recurrent implantation failure (RIF) or endocrine comorbidities, reducing the generalizability of the findings. Second, the random forest model in this study had a small sample size, affecting its stability and generalization. This issue was more evident in subgroup analyses, leading to increased standard errors and wider confidence intervals, thus raising statistical uncertainty. Subgroup analysis results are for trend identification only and are not definitive causal or predictive conclusions. In addition, the machine learning model was constructed and validated internally without external validation from other centers, which may limit its generalizability. Third, the number of high-quality embryos graded AB or BA was relatively limited, which may have led to a biased statistical result compared with BB and BC embryos; however, SHAP analysis confirmed the overall positive correlation between embryo quality and live birth, which was consistent with clinical experience. Forth, treatments in this study were primarily guided by clinicians’ judgment without standardized protocols or rigorous trial designs, limiting the ability to clearly link treatment effectiveness to miscarriage outcomes and reducing the study’s accuracy and reliability. Several improvements are proposed for further research. First, serial hCG doubling time data from day 14 to 21 post-transfer should be added to boost model performance. Second, negative results of immunotherapy cannot prove treatment ineffectiveness, This study merely proposes research hypotheses based on existing findings, further validation of the scientific validity and reliability of these hypotheses requires large-scale randomized controlled trials. Third, multicenter external validation is required prior to clinical implementation to verify model generalizability.

## 5 Conclusion

RSA can adversely affect the female reproductive system and represents a significant clinical challenge in the field of reproductive health. Among women with URSA undergoing euploid embryo transfer following PGT-A, β-hCG levels at day 14 post-transfer and embryo quality were robust, independent predictors of live birth. In contrast, empirical interventions and different types of luteal support showed no significant beneficial effects on pregnancy outcomes. The random forest model based on these two readily available clinical indicators showed favorable predictive performance. These findings highlight the importance of embryo selection and early post-transfer monitoring, while supporting the rational reduction of unnecessary clinical treatments. The prediction model established in this study may facilitate individualized clinical decision-making and improve the efficiency of reproductive management in this population.

## Data Availability

The original contributions presented in the study are included in the article/supplementary material. Further inquiries can be directed to the corresponding authors.
